# Cervical Spine Injuries: A Whole-Body Musculoskeletal Model for the Analysis of Spinal Loading

**DOI:** 10.1371/journal.pone.0169329

**Published:** 2017-01-04

**Authors:** Dario Cazzola, Timothy P. Holsgrove, Ezio Preatoni, Harinderjit S. Gill, Grant Trewartha

**Affiliations:** 1 Department for Health, University of Bath, Bath, United Kingdom; 2 Centre for Orthopaedic Biomechanics, Department of Mechanical Engineering, University of Bath, Bath, United Kingdom; 3 College of Engineering, Mathematics & Physical Sciences, University of Exeter, Exeter, United Kingdom; Leeds Beckett University, UNITED KINGDOM

## Abstract

Cervical spine trauma from sport or traffic collisions can have devastating consequences for individuals and a high societal cost. The precise mechanisms of such injuries are still unknown as investigation is hampered by the difficulty in experimentally replicating the conditions under which these injuries occur. We harness the benefits of computer simulation to report on the creation and validation of i) a generic musculoskeletal model (MASI) for the analyses of cervical spine loading in healthy subjects, and ii) a population-specific version of the model (Rugby Model), for investigating cervical spine injury mechanisms during rugby activities. The musculoskeletal models were created in OpenSim, and validated against *in vivo* data of a healthy subject and a rugby player performing neck and upper limb movements. The novel aspects of the Rugby Model comprise i) population-specific inertial properties and muscle parameters representing rugby forward players, and ii) a custom scapula-clavicular joint that allows the application of multiple external loads. We confirm the utility of the developed generic and population-specific models via verification steps and validation of kinematics, joint moments and neuromuscular activations during rugby scrummaging and neck functional movements, which achieve results comparable with *in vivo* and *in vitro* data. The Rugby Model was validated and used for the first time to provide insight into anatomical loading and cervical spine injury mechanisms related to rugby, whilst the MASI introduces a new computational tool to allow investigation of spinal injuries arising from other sporting activities, transport, and ergonomic applications. The models used in this study are freely available at simtk.org and allow to integrate *in silico* analyses with experimental approaches in injury prevention.

## 1. Introduction

The population incidence of acute spinal cord injury (SCI) is reported to be in the range of 16 to 40 cases per million [1] depending upon country, with the causes of these injuries ranging from motor vehicle collisions and community violence to recreational and workplace-related activities [2]. Whilst traffic collisions are associated with 50% of SCIs, an increasing proportion (currently 25%) of SCIs are due to sport and recreational activities [3]. This trend is mirrored by the demographic of SCI in the population, which is mainly represented by young men in their early thirties, who are more likely to be paraplegic, complete or incomplete, as an outcome [4]. The societal costs for traumatic spinal cord injuries are high, reported to be up to $9.7 billion ($US) per year in the USA [5], and up to £0.5–1 billion per year in the UK [6].

Musculoskeletal modelling is widely used in the field of biomechanics for the analysis and simulation of human motion, especially to investigate biomechanical variables that are not directly measurable through *in-vivo* experiments. For example, musculoskeletal models have been used to: inform surgical decisions through simulation [7, 8], analyse joint load [9], and identify motion patterns able to reduce injury risk in sporting activities [10]. Equally, musculoskeletal modelling can have many applications in the analysis of hazardous situations [11, 12] such as vehicle crashes or catastrophic injuries in sport, where direct measures on internal anatomical structures are not feasible or ethical.

*In vitro* experiments and *in silico* investigations have been widely performed in the field of transportation safety research [13, 14], but the primary mechanisms of acute spinal cord injuries are yet to be fully elucidated. A key limitation in many studies is the difficulty in replicating the contribution of neck and shoulder musculature to resisting external loads and, therefore, translation of the results to real world conditions has been limited.

Rugby Union (rugby) is a full contact sport that on rare occasions can result in serious spinal injuries, reported to range from 1-2/100,000 to 10/100,000 players per year [15, 16]. In the last few years there has been a specific focus on improving the safety of the sport, for example World Rugby (i.e. the international governing body) supported an injury prevention study that led to international scrum law changes [17], with the ultimate aim to reduce catastrophic injury occurrence in Rugby Union.

Similarly to transport applications, the biomechanical demands experienced during rugby activities, such as scrummaging and tackling, have been widely analysed [18–20], but the mechanisms of injury related to specific rugby activities are not yet fully elucidated. There is still an open debate in the literature regarding the primary injury mechanisms for cervical spine injury within rugby [21, 22], and both ‘buckling’ and ‘hyper flexion’ mechanisms have been proposed. Kuster, Gibson (21) argued that buckling is the predominant mechanism, whilst Dennison, Macri (22) stated that it is too early to draw that conclusion, and highlighted the need for *in silico* analyses, together with *in vivo* and *in vitro* tests, in order to provide new insights and identify viable routes for injury prevention.

We identified rugby activities as an appropriate proof of concept for the analysis of cervical spine loading and injury mechanisms using an *in silico* approach. During rugby scrummaging and tackling, the external load is applied through players’ shoulder girdle, feet and potentially other body regions, therefore, a full body model including detailed modelling of cervical spine and shoulder regions is required. Currently available ‘full-body models’ are limited to a combined trunk-upper limb segment or include welded scapuloclavicular joints. Besides, the majority of full body musculoskeletal models are used in gait analysis and locomotion simulations [23, 24], whilst those specifically focused on upper limb [25, 26], lumbar spine [27–29], and cervical spine [30] do not include either lower limbs or are kinematic-only models [31]. Therefore, the dynamic simulation of rugby activities necessitates a custom musculoskeletal model, which is purposely optimised for the analysis and simulation of rugby injury scenarios. Similar prerequisites are required also for the analysis of other injury scenarios, such as motor vehicles collisions and human collisions in physical activity settings.

Musculoskeletal models are often linearly scaled for segment dimensions [32, 33], but this procedure does not take into account subject- or population-specific musculoskeletal geometry and muscle parameters, which are fundamental to represent specific pathologies in clinical applications [34] and to describe the morphology of specific sub-populations. For instance, a rugby union Premiership forward player is on average 1.89 ± 0.07 m tall with a mass of 110.6 ± 7.7 kg [35], which is quite different from the population mean (height: 1.76 m, mass: 80.1 kg) [36] and more than 2 standard deviations greater than the anthropometrics used for ‘normal’ male subjects (height: 1.80 m, mass: 75.2 kg) [37] used in the creation of generic musculoskeletal models [37]. Thus, in the case of a rugby player, the body segment inertial properties and muscle functional parameters can differ considerably from those included in generic models.

The aim of the present study was to create, verify, and validate a generic full-body musculoskeletal model (‘Musculoskeletal model for the Analysis of Spinal Injuries’, or ‘MASI’) for *in silico* analyses of cervical spine loading during daily-living and sporting activities. A further population-specific version (the ‘Rugby Model’) of MASI that provides an *in silico* reproduction of a rugby forward player was customised and validated for the investigation of injury mechanisms during rugby activities.

## 2. Materials and Methods

The novel improvements embedded in MASI consist of i) a composite scapula-clavicular joint that provides the linkage between cervical spine, upper limb and the remainder of the body, and allows the application of multiple external loads, and ii) the inclusion of inertial and functional parameters to permit dynamic analyses. The Rugby Model was created to test the MASI in a specific sporting application, and differs from it only by the inclusion of inertial and muscle parameters more representative of rugby forward players.

### 2.1 Model structure

The ‘Musculoskeletal model for the Analysis of Spinal Injury’ (MASI) and Rugby Model were created in OpenSim (OpenSim 3.2, Simbios, Stanford, CA, USA) and Matlab software (Matlab 2013b, MathWorks, Natick, MA, USA), and share the same structure.

**Fig 1:**
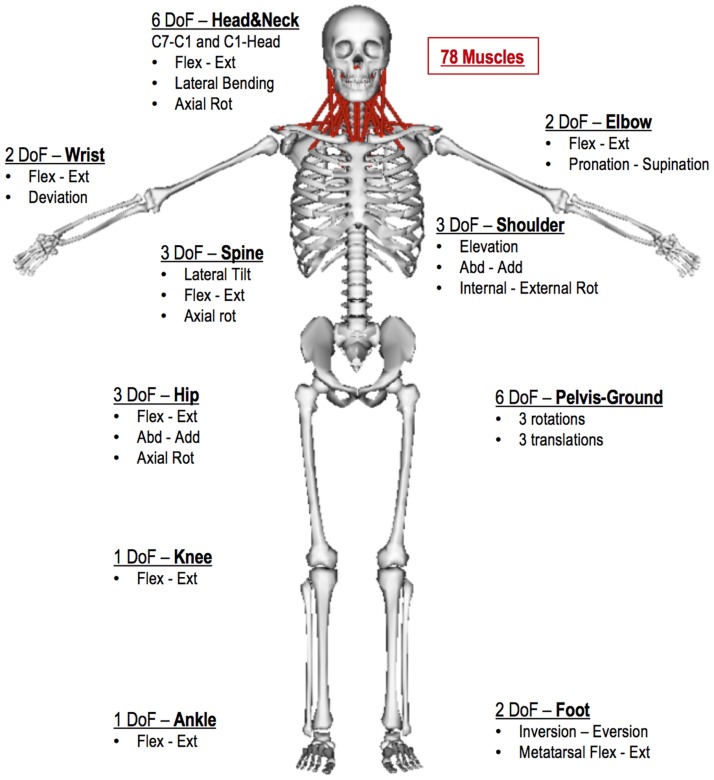
The degrees of freedom (DoF), and the anatomical segments of the Rugby Model. Body segments were divided into lower limbs (femur, tibia, talus, calcaneus and toe), pelvis, trunk, seven cervical spine vertebrae (C1-C7), scapula, clavicle, upper limbs (humerus, radius, ulna, hand) and head (skull and jaw). The 23 actuators of the model were associated to shoulder, elbow, spine, hip, knee, and ankle degrees of freedom.

MASI inherited the structure of the OpenSim head and neck model [30] which we embedded into a full body model (‘2354’, [23]), and was implemented to provide, for the first time, the linkage between cervical spine, upper limb, torso and lower limbs (Fig 1). MASI comprises 35 rigid anatomical segments, 78 upper and lower cervical muscles divided into 19 muscle groups, along with 23 torque actuators representing lower and upper limb muscles’ actions (Fig 1). Motion between body segments was permitted via 34 joints and 30 kinematic constraints. To incorporate the effect of upper limb position, a new scapula-clavicular joint (SCJ) (combining the joint motions of the acromioclavicular and sternoclavicular joints) was developed and included in the MASI, replacing the welded scapula-clavicular joint of the original head and neck model. The model had 43 degrees of freedom, though these were reduced to 37 by locking the metatarsophalangeal and wrist joints into the neutral position.

The creation, verification and validation of the MASI and Rugby Model were split into several phases to confirm firstly the performance of the scapula-clavicular joint, and secondly the cervical spine joints’ moment-generating capacity due to active and passive muscle forces.

### 2.2 Scapula-Clavicular Joint (SCJ)

The new SCJ was created in order to reproduce shoulder girdle motion in the scapular plane and include its effect on neck muscle behaviour while performing activities. Given the magnitude of the external load applied in some rugby activities, the SCJ was designed to minimise the number of intermediate massless bodies and kinematic constraints [38], which are integrated in more complex models [25, 26]. This modelling choice was driven by the need to minimise model complexity, as MASI and Rugby Model include a large number of kinematic constraints to reproduce neck movements.

**Fig 2:**
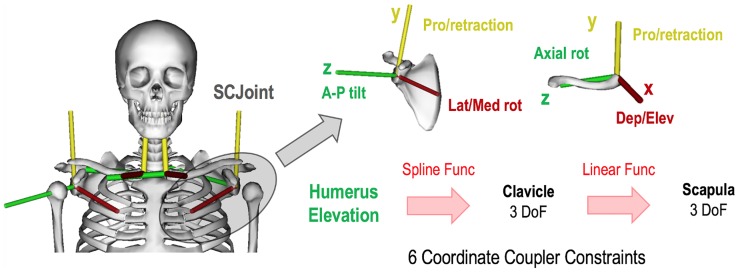
The SCJ’s reference systems on scapula (acromionclavicular) and clavicle (sternoclavicular) joints and their coupling functions driven by humeral elevation. The sternoclavicular joint origin on the sternal extremity of the clavicle and its reference system were designed to allow depression/elevation rotation about the x-axis, protraction/retraction rotations about the y-axis, and axial rotation about the z-axis. The motion of this joint is driven by the humeral elevation via 3 coordinate-coupler constraints based on spline functions. The acromionclavicular joint was designed with a glenoid-based reference system which allows lateral/medial rotation on x-axis, protraction/retraction rotations on y-axis, and anterior-posterior tilt on z-axis. The glenoid based system had the z-axis perpendicular to the glenoid plane, the y-axis directed superiorly toward the superior glenoid tubercle, and the x-axis directed anteriorly perpendicular to the other 2 axes. The motion of the acromionclavicular joint is driven by the sternoclavicular joint motion via 3 coordinate-coupler constraints based on linear functions.

The kinematics of the shoulder girdle is a composite of the motions of three bones (humerus, scapula, and clavicle) interacting at four articulations (glenohumeral, acromioclavicular, scapulothoracic, and sternoclavicular joints). The novel SCJ merges acromionclavicular and sternoclavicular joint motions, and its motion is a function of humeral elevation (Fig 2) as demonstrated in cadaveric and *in vivo* studies [39, 40]. The sternoclavicular joint was designed following the International Society of Biomechanics standard [41], whilst the acromionclavicular joint used a glenoid-based reference system [42]. The glenoid plane was defined based on landmarks on the glenoid rim, which were visually identified in OpenSim 3.2, and the glenoid-based system reference was reconstructed using Matlab software.

The SCJ was integrated in OpenSim through two custom joints that link the torso with the clavicle and the scapula with clavicle. Six mathematical functions (‘coordinate-coupled constraints’) were used to couple humeral elevation to clavicular and scapular motions. Permitted scapula and clavicle motions in the coronal, sagittal and transverse planes were obtained from *in vivo* data in the literature [40] and compared against *in vivo* kinematics measured using electromagnetic tracking system and bone pins [40, 42]. The coupling functions for the clavicle and scapula were extrapolated by correlating their motions relative to the humeral elevation, so that the sternoclavicular joint was driven by humeral elevation and the acromioclavicular joint by the sternoclavicular joint movements (Fig 2).

### 2.3 Inertial properties and neck muscles parameters

The MASI inherited body segment inertial properties mostly from the ‘2354’ model [23], whilst the cervical spine and head segments inertial properties were derived from *in vitro* data [43].

Body segment inertial properties for the Rugby Model were obtained from the literature [44–47] and combined with data from a DEXA scan (Hologic Discovery W, Bedford, MA; QDR software version 12.4.2) of a front row rugby player (height 1.84 m, 120.4 kg). The participant provided written informed consent prior to participation and ethical approval was obtained from the University of Bath Institutional Ethics Committee. The DEXA imaging allowed a more realistic distribution of the masses across the anatomical segments, while the local centre of mass locations and moments of inertia were personalised by inputting anthropometric data of the same rugby player into Yeadon’s mathematical inertia model [48] (Table 1).

**Table 1:** Mass distribution of the MASI derived from previous models and values from *in vitro* study [43]. Mass distribution of the Rugby Model calculated from DEXA values of a rugby forward player (1.84 m; 120.4 Kg). Masses are reported as percentage of total body mass. The principal moment of inertia (I_XX_, I_YY_, I_ZZ_) for the Rugby Model are shown in the las three columns of the table, and are expressed in kgm^2^.

SEGMENT	MASI MASS (%)	Rugby Model MASS (%)	Rugby Model I_xx_	Rugby Model I_yy_	Rugby Model I_zz_
**HEAD**	5.0	4.1	0.03328	0.01250	0.03589
**JAW**	0.6	0.6	0.04000	0.02000	0.04000
**TORSO**	26.2	22.3	1.22530	0.96260	2.18700
**CERV1**	0.7	0.6	0.03424	0.13697	0.03424
**CERV2**	0.9	0.8	0.03064	0.12254	0.03064
**CERV3**	0.6	0.6	0.03335	0.13338	0.03335
**CERV4**	0.6	0.6	0.03307	0.13229	0.03307
**CERV5**	0.6	0.6	0.03263	0.13051	0.03263
**CERV6**	0.7	0.7	0.03151	0.12605	0.03151
**CERV7**	0.9	0.8	0.03082	0.12327	0.03082
**PELVIS**	14.7	18.2	0.19204	0.16271	0.10816
**SCAPULA**	3.0	3.2	0.00124	0.00115	0.00137
**CLAV**	0.6	0.7	0.00024	0.00026	0.00004
**FEMUR**	9.3	9.9	0.17275	0.04528	0.18217
**TIBIA**	4.6	4.0	0.04650	0.00820	0.04650
**TALLUS**	0.1	0.1	0.00100	0.00100	0.00100
**CALCANEUS**	1.6	1.3	0.00179	0.00499	0.00525
**TOES**	0.3	0.2	0.00014	0.00028	0.00139
**HUMERUS**	2.5	3.4	0.02410	0.00831	0.02705
**ULNA**	0.8	1.0	0.00585	0.00122	0.00635
**RADIUS**	0.8	1.0	0.00585	0.00122	0.00635
**HAND**	0.6	0.8	0.00175	0.00108	0.00264

The maximum isometric force of the MASI neck muscles was initially checked by comparing the neck muscles’ moment-generating capacity of the OpenSim head-neck model (e.g. Vasavada model) [30] with measurements of healthy subjects’ neck strength from *in vivo* studies [49, 50]. However, Vasavada’s muscle parameters were obtained from *in vitro* cadaveric studies [51], and the neck muscles’ moment-generating capacity was found to be lower than healthy male subjects’ neck strength values [49, 50] (Table 2). This discrepancy was even more evident when compared with rugby players’ neck strength values [52], and particularly for the flexion moment generating-capacity, which was already identified as a limitation of the Vasavada model due to modelling assumptions [30]. Thus, we decided to scale the maximum isometric force of the Vasavada model in the MASI, aiming to match representative neck strength values for healthy male subjects [49, 50] for extension and lateral bending (Table 2). Two different scaling factors were used for extensors (i.e. 1.5) and flexors (i.e. 1.4); these scaling factors were calculated as the ratio of healthy male subjects’ neck strength [49, 50] to the maximum moment generated by the generic Vasavada model (Table 2). Similarly, the maximum isometric force of the Rugby Model’s muscles was scaled using different scale factors for extensors (i.e. 1.9) and flexors (i.e. 2.7) in order to match extension and lateral bending neck strength values of rugby players [52] from *in vivo* studies (Table 2). The modelling choice of specifically matching extension and lateral bending moment rather than flexion moment avoided the use of very high scaling factors that would unrealistically increase the passive moment of the flexors (i.e. passive stiffness).

**Table 2:** Neck strength values are reported from *in vivo* and *in silico* studies. Extension (*Ext*), flexion (*Flex*), axial rotation (*Axial Rot*), and lateral bending (*Lat Bend*) scaling factors are shown for adult rugby players (Rugby Front Row) and adult healthy males (Healthy Male). The scaling factors used to scale the MASI and Rugby Model neck muscles are calculated independently for flexors and extensors in order to match maximum extension and lateral bending neck strength values for a healthy male (Healthy Male) and a front row rugby player (Rugby Front Row), respectively. Head-Neck Model [30] was used as scaling reference, thus its scaling factor is 1.

Source	Ext—Flex (Nm)	Axial Rot (Nm)	Lat Bend (Nm)	Scaling Factors
**Head-Neck Model**				
*Vasavada et al*. *1998*	34.1–3.6	10.6	22.6	*1*
(*in silico* data)				
**Rugby Front Row**				*Ext (1*.*9)*
*Olivier et al*. *2008*	66–43	-	61	*Flex (11*.*8)*
(*in vivo* data)				*Lat Bend (2*.*7)*
**Healthy Male**				*Ext (1*.*5)*
*Fice et al*. *2014*	51–30	13	32	*Flex (8*.*3)*
(*in vivo* data)				*Axial Rot (1*.*2)*
				*Lat Bend (1*.*4)*
				*Rugby Front Row Player*
**Rugby Model**	72.3–15.9	19.7	58.5	*Ext (1*.*9)*
(*in silico* data)				*Flex (2*.*7)*
				*Healthy Male*
**MASI**	50.8–10.3	12.4	31.3	*Ext (1*.*5)*
(*in silico* data)				*Flex (1*.*4)*

### 2.4 MASI and Rugby Model verification and validation

Once the SCJ inertial properties and muscle parameters had been implemented, the models were verified and validated following the standards detailed in the literature for musculoskeletal modelling research [38]. The verification and validation procedures consisted of i) a kinematic validation, ii) a dynamic verification, and iii) a dynamic validation. The validated MASI and Rugby Model were then used to perform a forward simulation (i.e. computed muscle control) driven by *in vivo* neck functional movement kinematic data in order to simulate activation of the neck muscles and compare with experimentally-obtained muscle activity data.

Finally, the Rugby Model was used to run scaling, inverse kinematics, and inverse dynamic simulations during a rugby union scrum against an instrumented scrum machine, in order to test its application to rugby contact events.

#### 2.4.1 Kinematic validation

The kinematic validation was based on the comparison of model kinematics with experimental kinematics, and included i) the calculation of the range of motion of the SCJ, and ii) the analysis of moment arms of the muscles that originate on the scapula and clavicle bodies throughout the humeral range of motion.

The SCJ and humeral range of motion were calculated through an inverse kinematics procedure in OpenSim, driven by experimental *in vivo* data of a rugby forward player (age: 22 years, height: 1.77 m, mass: 88 kg) performing a humeral elevation in the scapular plane. The SCJ motion was compared with *in vivo* data of scapula and clavicle motion measured using bone pins [42], and *in silico* kinematics data generated using Holzbaur et al.’s previously validated OpenSim ‘Upper-Limb’ model [26]. *In silico* kinematics data were generated through an inverse kinematics simulation driven by the same experimental *in vivo* kinematic data of humeral elevation.

The inverse kinematics procedures enabled the calculation of the moment arms for the sternocleidomastoid and trapezius muscle groups using the Effective Torque Method [53], with an output comparison between the MASI and Holzbaur et al.’s model [26].

#### 2.4.2 Dynamic verification

The verification process aimed to verify that the custom SCJ adheres to the laws of physics, and in the case of the custom joint verification process, the main goal was to verify that the kinematic constraints did not generate any extra work, as constraint forces are often large but they are applied in non-movement directions so the power is zero. The power generated by the constraint was calculated as the sum of two elements: i) the scalar product of the external forces applied to the scapula and of the velocity of the centre of mass of the scapula; and, ii) the scalar product of the scapula generalised forces and corresponding generalised speed.

#### 2.4.3 Dynamic validation

The dynamic validation consisted of the determination of i) the passive neck stiffness, and, ii) the maximum net joint moment generated by the muscles throughout neck functional movements. The effect of humeral elevation on passive neck stiffness was also assessed.

The maximum net joint moment was estimated to occur when agonist muscles have maximal activation and antagonists have zero activation. The MASI and Rugby Model adopted a previously validated muscle-tendon model [54]. The passive neck stiffness was calculated when muscles had zero activation, and the neck motion generated under specific loading conditions was used to compare MASI and Rugby Model passive stiffness with *in vitro* and *in vivo* values [55]. Forward dynamics simulations generated neck movements (e.g. flexion-extension, axial rotation and lateral bending) through torque actuators that replicated the ‘2:4:2’ loading protocol (i.e. 2 Nm for flexion-extension and lateral bending, and 4 Nm for axial rotation applied to C2 vertebra) used in Miura, Panjabi (55). All the other bodies, except the scapula, the clavicle and the upper limbs, were locked in a stationary position The neck motion generated by the MASI and Rugby Model were considered acceptable if within 2 SD from *in vitro* values [55].

Maximal net joint moments were calculated during forward dynamics procedures that simulated a maximum isometric contraction with all the model’s bodies were locked and not free to move. The MASI’s and Rugby Model’s maximal net joint moments were considered acceptable if within 2 SD of *in vivo* reference data for maximal isometric neck contractions on dynamometers [49, 56].

#### 2.4.4 Neck muscles simulated activation during functional movement

Two different participants were recruited for the validation of muscle activation during neck functional movements using either the MASI or Rugby Model. Experimental data of full body kinematics, ground reaction forces, and neck muscles’ EMG of a healthy male subject (age: 64 years, height: 1.67 m, mass: 75 kg) and a male rugby forward player (age: 22 years, height: 1.77 m, mass: 88 kg) were collected during neck flexion, extension, lateral bending and axial rotation movements, respectively. The participants provided written informed consent prior to participation and ethical approval was obtained from the ‘Research Ethics Approval Committee for Health’ (REACH) of the University of Bath.

The trajectories of 68 reflective markers were measured through a 16-camera optoelectronic system (Oqus, Qualisys, Sweden; sampling frequency: 100 Hz), ground reaction forces were recorded via two Kistler force platforms (9287BA, Kistler Instruments Ltd, Switzerland; sampling frequency: 1000 Hz), and EMG signals from bilateral sternocleidomastoid, upper trapezius fibers and mid trapezius fibers were collected using Delsys Trigno (DelsysInc, Boston, Massachusetts, USA; sampling frequency: 2000 Hz). Raw EMG signals were rectified, filtered (Butterworth order 2, bandpass 10–500 Hz), and the envelope was created applying a 50-ms moving rectangular window. Experimental EMG signals were normalized using maximum voluntary contraction data collected using the procedure defined in Cazzola, Stone [57].

The MASI and the Rugby Model were scaled within OpenSim procedures to match the specific participant’s anthropometry based on the 68 motion analysis markers placed on anatomical landmarks (i.e. S1 Appendix). The OpenSim inverse kinematics algorithm solved for the joint angle time histories and then joint moments associated with participants’ motion were calculated using an inverse dynamics algorithm. A muscle control algorithm (computed muscle control or CMC) [58, 59] was then used to compute the muscle excitations required to track the kinematics produced by the inverse kinematics algorithm, by minimizing the sum of the square of muscle activations, while accounting for muscle activation and contraction dynamics [60]. The output of the CMC simulation was compared against the measured EMG data from the sternocleidomastoid and trapezius muscles during functional neck movements.

#### 2.4.5 Rugby Model application to rugby contact events: machine scrummaging simulation

A front row rugby player (age: 22 years, height 1.84 m, mass 120.4 kg) performed individual scrummaging trials against a strain-gauge instrumented scrum machine collecting external ‘shoulder’ forces at 500 Hz with feet positioned on force plates (9287BA, Kistler Instruments Ltd, Switzerland; sampling frequency: 2500 Hz). During scrum trials, a total of 68 reflective markers were positioned on the player (i.e. S1 Appendix) and tracked by a motion analysis system (Oqus, Qualisys, Sweden; sampling frequency: 250 Hz) and all data streams (kinematics, forces and EMG) were time-synchronised. The participant provided written informed consent prior to participation and ethical approval was obtained from the ‘Research Ethics Approval Committee for Health’ (REACH) of the University of Bath.

Joint kinematics (i.e. joint angles) and kinetics (i.e. joint net moment and joint reaction forces) at each individual cervical vertebra were obtained by using the Rugby Model during ‘Pre-Engagement’ and ‘Engagement’ phases of machine scrummaging. The Rugby Model was scaled to the participant’s anthropometric data, and inverse kinematics, Residual Reduction Algorithm (RRA), joint reaction forces analyses were run in OpenSim 3.2 (i.e. S1 Video). The RRA [61] was run to resolve the dynamic inconsistency between the measured kinematics and external loads applied to the system. The RRA routine was processed with force and kinematic data filtered through a 3rd-order low-pass bidirectional Butterworth filter at 12 Hz.

The joint reaction forces analysis was run without considering neck muscles contribution, and therefore calculating the joint reaction forces as follows:

$$R_{j}=m_{j}a_{j}-\left(\sum F_{ext}+\sum F_{const}+R_{j+1}\right)$$

where R_j_ is the reaction force at joint ‘j’, m_j_ is the mass of segment ‘j’, a_j_ is the centre of mass acceleration of a segment ‘j’, F_ext_ is the sum of all external force applied to a segment ‘j’, F_const_ is the sum of all constraint forces applied to a segment ‘j’ and R_j+1_ is the reaction force at an adjacent segment ‘j+1’.

## 3. Results

### 3.1 Kinematic validation: Scapula-Clavicular Joint (SCJ) and moment arms

**Fig 3:**
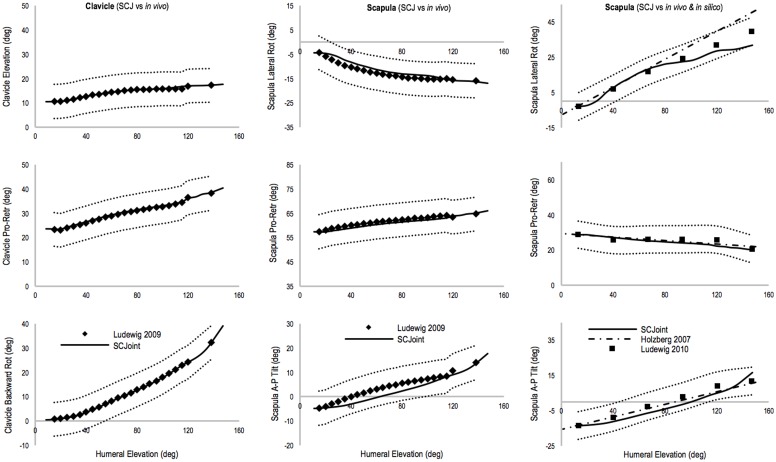
Sternoclavicular (SC) (left column) and acromioclavicular (AC) (middle and right columns) motions during humeral elevation. The black diamonds in the left and middle columns graphs represent SC and AC kinematics during *in vivo* measurements [40], whereas black solid lines are the respective SC and AC kinematics generated by the SCJ. The SC and AC joints angles (black solid line) are within 2SD from *in vivo* values [40] (black dotted lines). In the right column graphs, the AC kinematics generated by SCJ is compared with another *in vivo* study and an *in silico* study. The AC motions generated by the SCJ (black solid line) is within 2SD (black dotted lines) from *in vivo* studies in the literature [41] (black squares) and comparable to the output of the Holzbaur’s model [26] (black dash-dot line). The glenoid reference system of the scapula was roto-traslated in order to express scapula motion with respect to the acromioclavicular joint reference system, and compare it with Ludewig, Hassett (42) and Holzbaur, Murray (26) studies.

The scapula and clavicle movements throughout the humeral range of motion were within two standard deviations of experimental measurements from *in vivo* [40, 42] studies (Fig 3). The sternoclavicular (SC) and acromioclavicular (AC) motions modelled through the SCJ were similar to experimental kinematics measured in the Ludewig, Phadke (40) study over the 20–140° range of humeral elevation: the RMS deviation of SC motions varied from 0.1° to 0.3°, whilst the RMS deviation of AC motions varied from 2.5° to 3.6°.

The AC motions generated by the SCJ were also compared with a previous *in silico* study [26]: the RMS deviation from Holzbaur’s model across the range of humeral elevation was higher for scapula lateral rotation (12.4°), than protraction-retraction (1.2°) and anterior-posterior tilt (3.3°) motions. The higher deviation for lateral rotation was due to a different behaviour between the SCJ and Holzbaur’s model for humeral elevation values greater than 90° (Fig 3). However, the same AC motions compared with *in vivo* measurements [42] showed a low RMS deviation across lateral rotation (3.4°), protraction-retraction (1.8°), and anterior-posterior tilt (3.5°).

**Fig 4:**
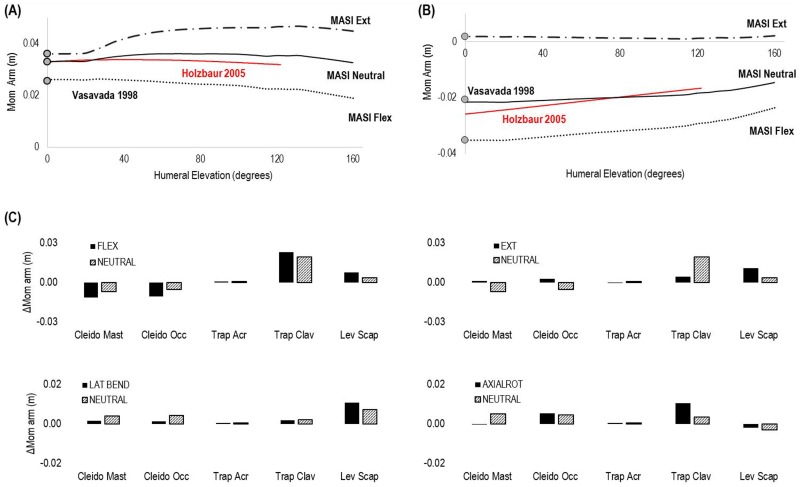
Moment arms of elevator scapulae (A) and sternocleidomastoid (B) muscles throughout humeral elevation when the neck was fully extended (Ext—black dashed and dotted line), flexed (Flex—black dotted line) and in neutral position (Neutral—black solid line). Vasavada’s (grey circles) and Holzbaur’s moment arms (red solid line) across humeral elevation are compared with MASI’s moment arms when the neck is at neutral position. (C) Muscles’ moment arms maximum changes of flexion (FLEX), extension (EXT), lateral bending (LAT BEND) and axial rotation (AXIAL ROT) moments during humeral elevation when the neck is fully flexed, extended, laterally bent, axially rotated and in neutral position (NEUTRAL). Positive values represent an increase whilst negative values a decrease of the moment arm across humeral elevation.

The moment arms of the muscles having origin or insertion points on the clavicle and scapula bones during humeral elevation in the scapular plane (Fig 4) equalled the previous Vasavada model estimations when the neck and the humerus were in neutral position (Fig 4a and 4b), and were comparable with Holzbaur’s model (Fig 4a and 4b) across the range of neck motion and humeral elevation.

The MASI model had muscle moment arms which varied over humeral elevation, which is key for correctly estimating the contribution of neck muscle forces during activities involving upper limb motion. The sternocleidomastoid muscle group mainly showed a decrease (~0.01 m) of flexion moment arm in neutral and fully flexed head poses through the range of humeral elevation, and an almost negligible decrease (~0.005 m) in the axial rotation moment arms when the neck was axially rotated (Fig 4c). The flexion moment arms of the superior fibres of trapezius (i.e. Trap Clav in Fig 4c) mainly increased (~0.020 m) during humeral elevation when the neck was fully flexed and in neutral position (~0.017 m), whilst an extended position minimised this change. Levator scapulae lateral bending moment arms increased during humeral elevation when the neck was laterally bent (~ 0.01 m) and in neutral pose (< 0.01 m), whilst its extension moment increased in fully extended pose (Fig 4c).

### 3.2 Dynamic verification and validation

**Fig 5:**
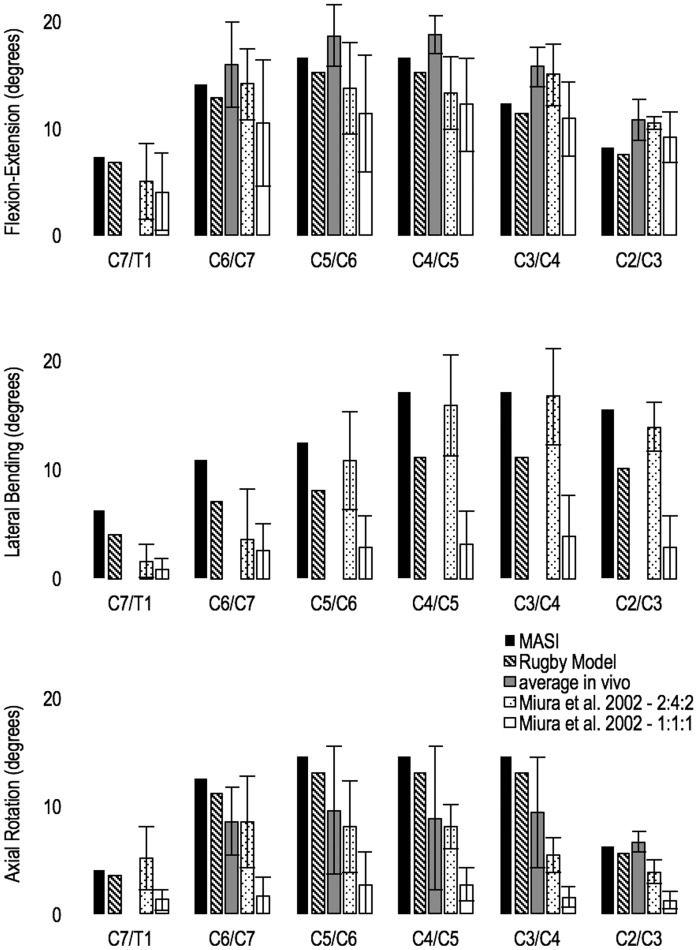
Neck joints motion under a 2 Nm (flexion-extension and lateral bending) and 4Nm (axial rotation) load applied at C2 vertebra. MASI’s neck motion (black bars) and Rugby Model’s neck motion (black downward diagonal bars) are compared with an average value from different *in vivo* studies (grey bars) and two protocols of an in vitro study: ‘2-4-2’ protocol (white dotted bars) and ‘1:1:1’ protocol (white bars). Average *in vivo* values were not available for lateral bending individual joint motion (middle graph).

The forward simulations confirmed that the power generated by the SCJ kinematic constraint was nil, therefore the constraints were workless. The passive joint moments generated during neck movement and humeral negative elevation (Fig 5a) were comparable with *in vitro* data [55], in the region of 2–4 Nm.

The maximal net joint moments generated by the MASI were comparable with healthy male subjects’ neck strength for extension and lateral bending movement, whilst flexion moment-generating capacity of the model resulted more realistic than Vasavada’s model, yet still 3 times lower than in vivo values previously reported (Table 2). For the Rugby model, the maximal net joint moments generated by activated neck muscles during extension and lateral bending were comparable with rugby forward players’ neck strength in vivo values, whilst flexion moment-generating capacity was 3 times lower (Table 2).

The passive neck stiffness of MASI was generally within 2SD from Miura, Panjabi (55) *in vitro* values and the average of *in vivo* values [62–67] in flexion-extension, lateral bending and axial rotation motions (Fig 5). During flexion-extension, the lower-middle cervical spine (C7/T1-C4/C5) showed a higher level of agreement with in vivo values than in vitro values, as joint motion was slightly higher (5 to 10%) than *in vitro* values. On the contrary C2/C3 joint showed higher stiffness and lower flexion-extension motion than both *in vivo* and *in vitro* values. Passive neck stiffness in lateral bending was generally similar to *in vitro* values for middle-upper spine (C5-C2), except C7/T1 and C6/C7 joints that showed values greater than 2SD different. Neck passive stiffness in axial rotation was generally lower (i.e. higher degree of motion) than *in vitro* values, and more in line with *in vivo* values.

The neck passive stiffness of the Rugby Model followed the same pattern shown by the MASI, but the degree of motion across all the neck movements was lower than MASI (~8% in flexion-extension and ~11% in axial rotation), in particular for lateral bending (~30%) due to higher stiffness emanating from the doubling of maximal isometric muscle forces. Concomitantly, *in vivo* values for rugby forward player’s cervical spine range of motion were found lower than for healthy males [68].

The passive neck moment in flexion and lateral bending motion, when the humerus was maximally elevated, increased respectively to 5 Nm and 4 Nm due to the increased length of the levator scapulae and trapezius muscles. Passive neck moment in axial rotation at maximal humeral elevation was lower than 2 Nm.

### 3.3 Neck muscles activation during functional movements

**Fig 6:**
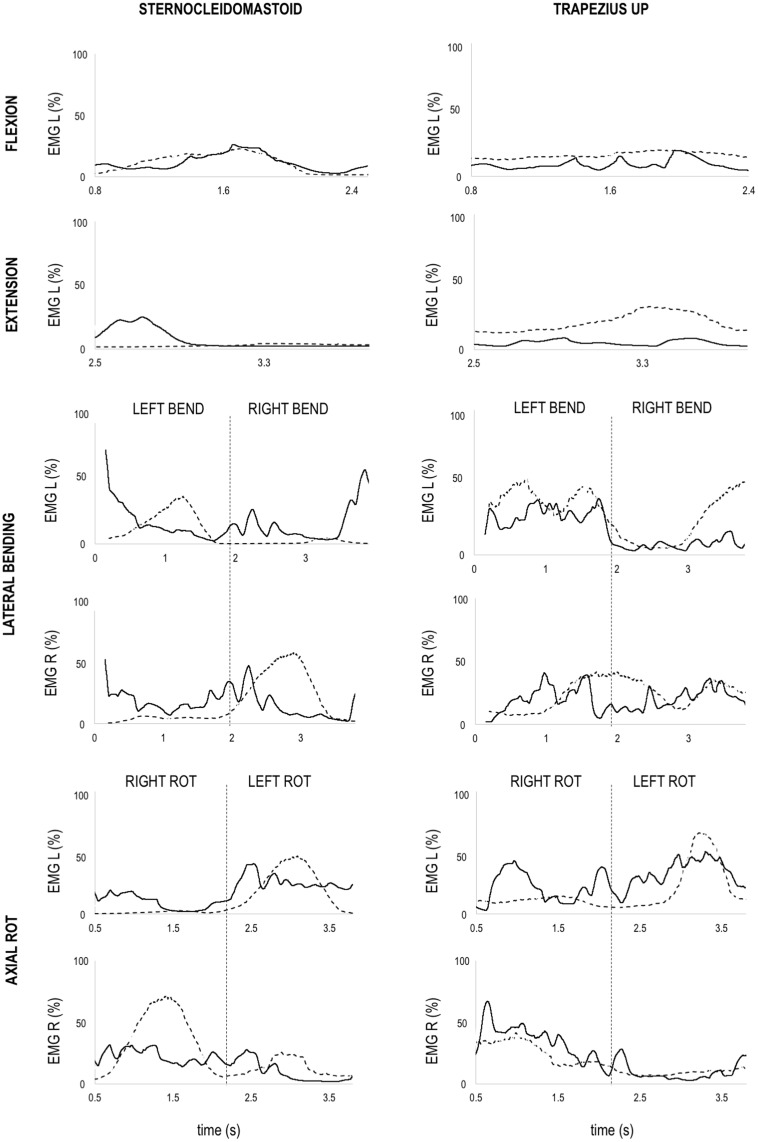
Rugby Model simulated neck muscle activation. Simulated muscle activation from computed muscle control (solid black line) and experimental EMG (dashed black line) of sternocleidomastoid (left column) and upper trapezius (right column) muscles during flexion, extension, lateral bending (right and left bending) and axial rotation (right and left rotation). Experimental EMG signal were normalized using maximum voluntary contraction data and defined between 0% and 100%. Simulated activations are defined between 0% (no activation) and 100% (full activation).

**Fig 7:**
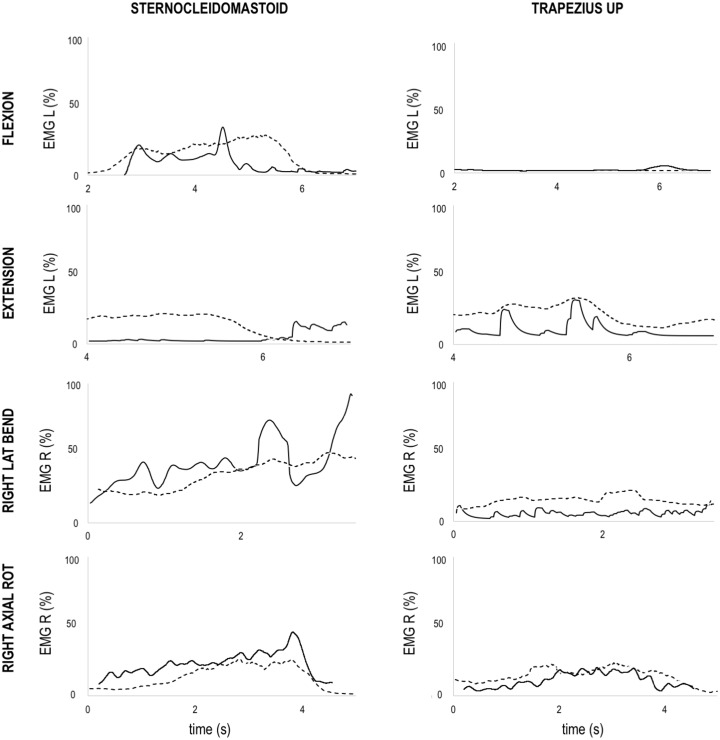
MASI simulated muscle activation. Simulated muscle activation from computed muscle control (solid black line) and experimental average rectified EMG (dashed black line) of sternocleidomastoid (left column) and upper trapezius (right column) muscles during flexion, extension, lateral bending (right bending) and axial rotation (right rotation). Experimental EMG signal were normalized using maximum voluntary contraction data and defined between 0% and 100%. Simulated activations are defined between 0% (no activation) and 100% (full activation).

Using qualitative inspection, the simulated activation of trapezius muscles showed a similar pattern and activation level in comparison with the recorded EMGs across the neck movements analysed (Figs 6 and 7).

The sternocleidomastoid muscles activations simulated by using the Rugby Model were generally comparable with experimental EMGs, except for the left sternocleidomastoid during extension and lateral bending (Fig 6). During neck extension the left sternocleidomastoid was activated at the beginning of the movement, although experimental EMG showed an almost nil activation across the movement (Fig 6). However, the sternocleidomastoid activations simulated by using the MASI showed more consistent pattern compared with the experimental EMG (Fig 7).

During lateral bending, the activation of the left sternocleidomastoid simulated by using the Rugby Model showed an anticipated high activation in left bending, and a late high activation during right bending with respect to experimental EMG (Fig 6). However, the same activation pattern was not present when sternocleidomastoid activation was simulated by using MASI (Fig 7).

### 3.4 Biomechanical load experienced during machine scrummaging

**Fig 8:**
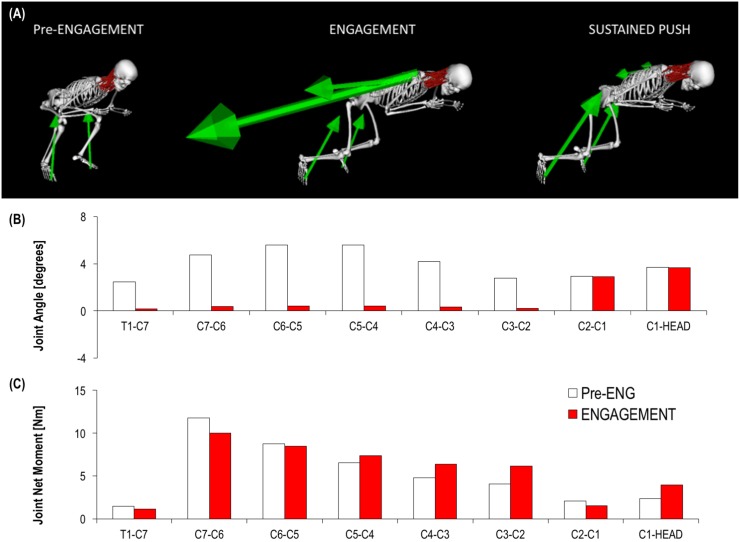
(A) The three main phases of a rugby scrummaging activity: pre-engagement phase, engagement phase, and sustained-push. (B) Average flexion-extension joint angle across cervical spine joints during ‘Pre-Eng’ and ‘Engagement’ phases. Extension motion is positive in the graph. (C) Flexion-extension joint moment values during pre-engagement and engagement phases across the cervical spine vertebral joints. Extensor moment is positive in the graph. The graph shows the decreasing pattern of extensor moment from C7-C6 joint to C1-Head, which is mainly due to resist the flexion moment generate by the gravity force.

The cervical spine was in an extended position at T1-C7 to C2-C3 joints prior to contact but then underwent a flexion motion during impact, whereas the C2-HEAD joint remained extended throughout the engagement phase (Fig 8b). This cervical spine motion caused a flattening of cervical spine curvature during the engagement phase.

The biomechanical load experienced at vertebral joint level is representative of the joint moments and forces that the neck muscles and other passive structures have to overcome or resist during a machine scrummaging trial (Fig 8a). The joint net moments calculated across all the vertebral joints were quite low and representative of the moment needed to support the head and cervical vertebra weight (Fig 8c).

**Fig 9:**
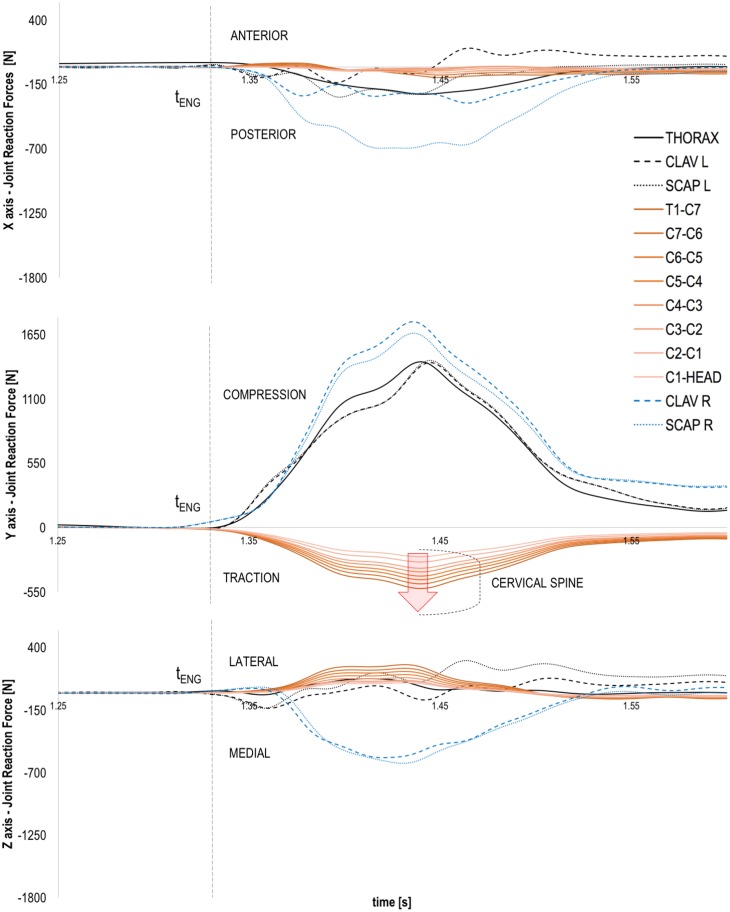
Typical joint reaction force traces of scapula, clavicle, thorax (thoracic and lumbar spine plus ribcage) and cervical spine bodies during a machine trial. The graph at the top shows the joint reaction force in anterior-posterior direction (X axis), whilst the central and bottom graphs show respectively the joint reaction forces in the longitudinal (Y axis) and mediolateral (Z axis) directions. The time of real engagement (tENG) is highlighted by the dashed vertical line. The red arrow highlights the increasing pattern of tensile load at cervical spine level.

The peaks of joint net forces at cervical spine level were higher in longitudinal direction (Y axis) than in mediolateral (Z axis) and anteroposterior (X axis) directions. Longitudinal joint reaction forces decreased from lower to upper cervical joints (Fig 9) ranging from ~-500 N (T1-C7) to ~-200 N (C1-HEAD). The right and left acromioclavicular joints showed the highest joint reaction forces in both longitudinal (~1700 N) and mediolateral (~600 N) directions (Fig 9). The cervical spine joint reaction forces in mediolateral directions ranged from ~80 N (T1-C7) to ~250 N (C1-HEAD). Overall, the joint reaction analysis in longitudinal direction showed a ‘distraction’ pattern of force (i.e. tensile load) at cervical spine level. This is due to the fact that during the engagement phase the external load is mainly acting at the shoulders with the effect that the applied force is pushing back the trunk whilst the neck and head bodies are accelerated forward by the inertia of the preceding movement.

## 4. Discussion

The MASI and the Rugby Model are novel musculoskeletal models created for injury mechanism investigations, and its final aim is to provide internal measurements for cervical spine structures that cannot be collected experimentally because they are too invasive or not ethically acceptable. The MASI is an *in silico* representation of a healthy male subject and the present validation demonstrates that it is suitable for investigating cervical spine loading through both inverse and forward simulations. The key novel aspects of the presented models with respect to previous head and neck models [30] are i) the integration of the head/neck model with a comprehensive full body model, ii) the implementation of the scapula-clavicular joint (SCJ) for the application of external loads on the shoulder girdle, and iii) the inclusion of population-specific inertial properties (e.g. Rugby Model) and muscle parameters to permit dynamic analyses.

The analysis of cervical spinal injuries mechanisms is one of the grand challenges of injury prevention research, and we identified rugby union activities as a perfect proof of concept due to impact loading being applied to the shoulder/neck region and distinctive anthropometric properties of the participants. Thus, we created and validated the Rugby Model, which is a population-specific version of the MASI, and includes a set of inertial properties and muscles parameters to reproduce the features of a rugby forward player. The Rugby Model was used to analyse for the first time the cervical spine loading in rugby-related contact events (i.e. inverse simulation of machine scrummaging) (i.e. S1 Video). The inclusion of lower limbs and shoulder girdle allowed to application of the model to situations that require the inclusion of multiple external loads, such as rugby scrummaging.

The MASI and Rugby Model provide validated shoulder girdle and cervical spine kinematics, and allow estimates of the moment arms during coupled neck and arm movements. From a dynamic perspective, forward simulations confirmed their validity at reproducing *in vivo* maximal net joint moments and comparable *in vitro* [55] cervical spine passive stiffness.

The custom scapula-clavicular joint (SCJ) was created for conducting kinetic analysis of rugby activities during which the humeral and scapula-clavicular movements and external loading through these structures play an important role. The SCJ successfully simulates sternoclavicular and acromioclavicular motions in comparison with experimental values from *in vivo* [40, 42] and *in silico* [26] studies (Fig 3). This comparison also showed that SCJ and Holzbaur’s models, at humeral elevation values larger than 90°, generates respectively lower and higher scapular lateral rotation values with respect to *in vivo* measurements. However, the SCJ deviation is within 2SD from *in vivo* data, whilst Holzbaur’s model exceeds 2SD for humeral elevation higher than 120°. This is due to the different regression equations and kinematic constraints used to drive the models, which can affect trapezius and levator scapulae moment arms at large humeral elevation.

The SCJ allows the estimation of neck muscles moment arms during combined shoulder and neck movements (Fig 4). This improves the moment-generating capacity of the model [69], and therefore the estimation of individual neck muscle forces for the muscles that have origin or insertion points on the clavicle and scapula bones. During a rugby scrum or rugby tackle a player is exposed to external loads of 2–4 kN applied on his/her shoulders [18, 19, 70], and neck movements are typically performed while the humerus is elevated in the scapular or coronal planes. In these postures, moment arms are dependent on humeral elevation, and their values can affect static optimisation algorithms. A further refinement would be the implementation of an algorithm for representing the 3D interaction of muscle surfaces, allowing moment arms to be better estimated using wrapping surfaces based on subject-specific data [69, 71], as neck muscle geometry of rugby players might considerably differ with respect to healthy subjects due to hypertrophy as a result of muscle conditioning. In its current form the novel SCJ is not intended to replace shoulder models based on closed-loop kinematic chains [25, 72–74], and optimised to provide accurate simulations for clinical applications [75]. Indeed, as in previous shoulder models [26, 76], the SCJ is driven by regression equations that do not change according to participants’ morphological characteristics, and does not include a constraint reproducing the gliding plane of the scapulothoracic joint. The SCJ is only intended to reproduce shoulder kinematics during humeral elevation in the scapular plane, which is the most representative elevation plane for the analysis of rugby activities.

The MASI and Rugby Model allowed satisfactory replication of cervical spine passive and active behaviour in healthy subjects and rugby players. However, the passive neck stiffness calculated in this study is mainly representative of passive muscle forces, as the neck kinematic constraints can only partially replicate the stiffness from other passive structures. This is a limitation of MASI and Rugby Model and future inclusion of ligaments and intervertebral discs will be beneficial for better reproducing stiffness values in lateral bending and axial rotation.

The neck muscle activations simulated by using the Rugby Model and MASI were generally comparable with the experimental EMGs during neck functional movements (Figs 6 and 7), although the sternocleidomastoid showed different activation patterns when not used as primary movers of neck and head bodies (see left sternocleidomastoid during extension and right lateral bending in Fig 6). In these movements, the sternocleidomastoid is mainly used to decelerate neck extension via eccentric contraction and control head motion, which is a difficult task to simulate with current optimisation functions. In fact, muscle activations are dependent on the objective function used to solve the redundancy problem, and an optimised function for neck motion analysis still needs to be found. In this study, we minimised the sum of the neck muscle forces, but different objective functions that includes the use of synergies, or EMG driven simulations should be explored to provide more realistic results. Therefore, our simulation results demonstrate model suitability for the calculation of muscle forces during functional activities rather than providing benchmarking values. However, the same validation procedure provided positive results using the MASI driven by *in vivo* data of a healthy male subject (Fig 7), demonstrating that the model’s output is appropriate even with different inertial parameters and muscle scaling factors. Further validation procedures are still needed for reconstructing muscle activations during specific rugby activities such as scrummaging, in which the combination of small upper body movements and muscle co-contractions [57] may affect the effectiveness of the current static optimization redistribution approach based on the minimisation of the sum of muscles activation squared or sum of muscles force.

The new set of inertial parameters of the Rugby Model derives from a rugby front row player’s DEXA scan, with segment centre of mass position and moments of inertia personalised to the individual using a mathematical inertia model [48]. This combined approach is beneficial for generating a musculoskeletal model that better replicates rugby players’ anthropometry when medical imaging is not readily available, and therefore improves linear-scaling and inverse dynamics results with respect to a generic OpenSim model (e.g. healthy male of 1.8 m and 75.16 kg). The proposed method does not substitute for a subject-specific scaling method based on medical images (e.g. MRI and CT scans), which is still considered the gold standard to estimate muscle origin and insertion points [24] and body segment inertial properties [77]. Also, optimisation technique methods [78] or a scaling method based on the combination of 3D body-surface and DEXA scans would provide a better estimation of body geometry [79], a specific density profile [80] and would theoretically produce more reliable simulation outputs.

The Rugby Model includes population-specific muscle strength parameters derived from *in vivo* studies, which becomes fundamental to generate realistic simulations when muscle physiological cross-sectional area (PCSA) and maximum isometric force values differ considerably from generic data. Individualisation of muscle parameters can be performed using simple scaling methods [81] or more complex imaging-based methods [34]. Bolsterlee, Vardy [82] demonstrated that a uniform scaling routine provided reliable prediction of a shoulder model’s strength, and the scale factors can either be obtained from muscle volume data or maximum strength measurements. Given the modelling assumptions of the Vasavada model (i.e. potentially different joint centre of rotation during flexion and lack of infrahyoid muscles in the model) [30], the maximum isometric force of the Rugby Model’s neck muscles was scaled using two scaling factors, both obtained from maximum strength measurements of rugby forward players (Table 2).

The same approach was used for the MASI and allowed a better match to *in vivo* neck strength values of a healthy male subject with respect to previous models [30]. However, the neck flexion moment generating-capacity was still three times lower that *in vivo* values, and this was a limitation of the Vasavada model due to modelling assumptions [30] that was inherited by both the Rugby Model and MASI.

The construction of subject-specific models is still needed for more accurate simulations, but the presented models can already provide the biomechanics community with a computational tool to analyse rugby activities (i.e. the Rugby Model) or more generic applications where cervical spine loading is of interest (i.e. the MASI). Both models are freely downloadable from SimTK (https://simtk.org/home/csibath).

Further modelling developments will include the replacement of neck kinematic constraints with contact models [28, 83] and the inclusion of EMG driven simulations [84] for the estimation of muscle forces. Future application of the models will firstly include the simulation of injurious events, and the analysis of neck muscle contribution to cervical spine loading during rugby activities, such as scrummaging and tackling. This first step will allow us to further optimise the model for analysis of collisions in sports such as American Football and Ice Hockey, as well as the analysis of cervical spine injuries during motor vehicle collisions.

## Supporting Information

S1 Appendix Experimental Set-up. Description of the experimental set up and marker set used in the study.
(DOCX)

S1 Video Machine Scrummaging inverse simulation. Simulation of a machine scrummaging trial from three different views. Green arrows represent the force applied to the rugby player during the different phases of a machine scrummaging trial.
(M4V)
