# Supplementary material for: Cervical Spine Injuries: A Whole-Body Musculoskeletal Model for the Analysis of Spinal Loading
Source: PLoS One. 2017 Jan 4;12(1):e0169329. doi: 10.1371/journal.pone.0169329 (PMC5214544; doi:10.1371/journal.pone.0169329)
Supplement: S1 Appendix — Description of the experimental set up and marker set used in the study. (DOCX) [file pone.0169329.s001.docx]

# Appendix

### Experimental Set-Up

A marker set of 68 markers was integrated into the MASI and the Rugby Model (S1 Figure). The anatomical landmarks used for markers placement are shown in S1 Table 1. Clusters of 4 markers were used to track participants’ movements during dynamic trials for thigh, shank and arm anatomical segments.


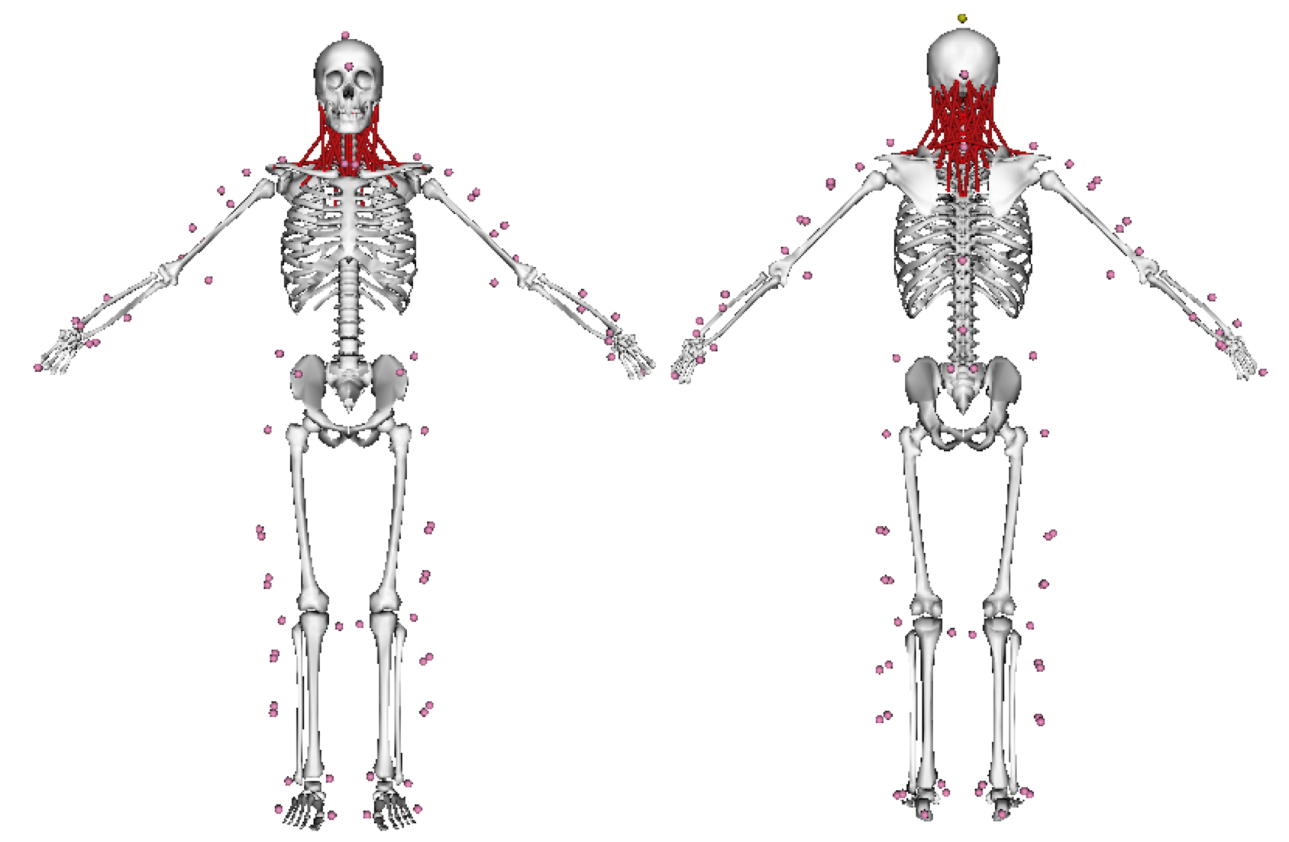


S1 Fig. MASI and Rugby Model including reflective markers position.

Table 1. Description of the biomechanical model represented in S1 Fig: segments and markers.

| **Segments** | **Description** |
| --- | --- |
| Head | Nasium, Vertex and Occipital Bone. |
| Trunk | Markers applied on C7, T8, L3. |
| Right and Left Scapula | No markers applied. Size estimated using clavicle markers (acromion and sternum). Movement tracked by upper arm motion thorough the scapuloclavicular joint. |
| Right and Left Upper-Arm | 4 markers clusters. |
| Right and Left Fore-Arm | Medial and lateral elbow and Ulnar and Radial styloid processes. |
| Right and Left Hand | Ulnar and Radial styloid process and on the hand, just below the third metacarpus. |
| Pelvis | Bilaterally, markers on PSISs, ASISs and iliac crests. |
| Right and Left Thigh | Greater trochanter, lateral and medial knee condyles and 4 markers cluster. |
| Right and Left Shank | Medial and lateral knee, medial and lateral malleolus and 4 markers cluster. |
| Right and Left Foot | Medial and lateral malleolus, heel, 1^st^ metatarsal and 5^th^ metatarsal. |
